# Trends and distribution of external radiation therapy facilities in Japan based on Survey of Medical Institutions from the Ministry of Health, Labour and Welfare

**DOI:** 10.1093/jrr/rrae014

**Published:** 2024-04-11

**Authors:** Takahiro Aoyama, Yutaro Koide, Hidetoshi Shimizu, Tomoki Kitagawa, Tohru Iwata, Shingo Hashimoto, Hiroyuki Tachibana, Takeshi Kodaira

**Affiliations:** Department of Radiation Oncology, Aichi Cancer Center, 1-1 Kanokoden, Chikusa-Ku, Nagoya, Aichi 464-8681, Japan; Department of Radiation Oncology, Aichi Cancer Center, 1-1 Kanokoden, Chikusa-Ku, Nagoya, Aichi 464-8681, Japan; Department of Radiation Oncology, Aichi Cancer Center, 1-1 Kanokoden, Chikusa-Ku, Nagoya, Aichi 464-8681, Japan; Department of Radiation Oncology, Aichi Cancer Center, 1-1 Kanokoden, Chikusa-Ku, Nagoya, Aichi 464-8681, Japan; Department of Radiation Oncology, Aichi Cancer Center, 1-1 Kanokoden, Chikusa-Ku, Nagoya, Aichi 464-8681, Japan; Department of Radiation Oncology, Aichi Cancer Center, 1-1 Kanokoden, Chikusa-Ku, Nagoya, Aichi 464-8681, Japan; Department of Radiation Oncology, Aichi Cancer Center, 1-1 Kanokoden, Chikusa-Ku, Nagoya, Aichi 464-8681, Japan; Department of Radiation Oncology, Aichi Cancer Center, 1-1 Kanokoden, Chikusa-Ku, Nagoya, Aichi 464-8681, Japan

**Keywords:** external radiation therapy, high-precision radiation therapy, radiotherapy facility, population ratio disparity, density rate disparity, Survey of Medical Institutions

## Abstract

This study aimed to explore the distribution of external radiation therapy (RT) facilities, the status of related device installations and the adoption of high-precision RT using Survey of Medical Institutions from the Ministry of Health, Labour and Welfare in Japan. Analysis, categorized by the hospital size and prefecture, provides specific insights into the trends in treatment facility healthcare capabilities. Data on the number of RT facilities, high-precision RT facilities, RT devices and treatment planning systems (TPS) categorized by the number of beds and prefecture from 1996 to 2020 were analyzed. In addition, the study examined the correlation between the high-precision implementation rate and the number of TPSs or radiation oncologists and other medical staff. High-precision RT exceeded 95% in large facilities (800+ beds) but remained <50% in medium-sized facilities (300–499 beds). In a prefecture-by-prefecture analysis, calculation of the maximum–minimum ratio of RT facilities per million population and per 30 km^2^ revealed a disparity of 3.7 and 73.1 times in the population ratio and the density ratio, respectively. Although a correlation was found between the number of TPSs per RT device or the number of medical physicists per million population and the rate of high-precision RT implementation, no correlation was found among other professions. Detailed analysis based on the hospital size and prefecture provided more specific information on the medical functions of RT facilities in Japan. These findings can potentially contribute to the future development of RT, including the standardization of treatment techniques and optimal resource allocation.

## INTRODUCTION

Radiation therapy (RT) is an essential treatment modality in cancer management, and the proper placement of treatment techniques and related equipment directly influences healthcare standardization and improvement. The situation surrounding RT has changed, particularly, over past two decades, with remarkable advances resulting from improved treatment techniques and the development of new treatment devices.

In Europe and North America, approximately 50% of patients with cancer receive RT and > 70% of facilities can perform intensity-modulated radiation therapy (IMRT) [1–3]. In South Korea, the utilization rate of IMRT exceeded that of 3D-conformal RT in 2019 [4]. However, disparities in utilization between metropolitan and non-metropolitan areas have been noted, indicating the need to understand and correct these disparities through national surveys. According to reports for Japan, the utilization rate of RT for all patients with cancer is 23.7%, while the utilization rate of IMRT for all irradiations is 16.8% [5]. These rates are considerably lower than those observed abroad. However, due to the aging of the Japanese population, it is expected that this demand will increase in the future. Therefore, comprehensively investigating the domestic situation, examining the equipment deployment status and evaluating the implementation of RT treatments is essential to address this increasing demand and support future development. To efficiently achieve these goals, cross-sectional, long-term and highly accurate fixed-point surveys are required. In particular, large-scale and comprehensive data on the entire country, rather than data on individual hospitals or regions, are required.

The Japanese structure survey of radiation oncology [5–22], published by the Japanese Society of Radiation Oncology (JASTRO), is an important long-term, nationwide survey for understanding the deployment of RT devices and implementation status of RT treatment. However, the survey response rate is only 80–90% (87.2% in 2019) and nationwide RT data are not entirely captured. Conversely, the Survey of Medical Institutions Database, published by the Ministry of Health, Labour and Welfare (MHLW) [23], better meets these criteria. With a high response rate of 99.9% in 2020 and continuous surveys conducted for >25 years since its initiation in 1996, this is considered the most reliable data source for revealing the actual state of RT and distribution and status of medical facilities. Although some reports used Survey of Medical Institutions that address regional disparities in healthcare resources [24, 25] and the deployment status of the equipment and personnel in the field of radiological diagnostics [26, 27], to date, reports have not specifically investigated treatment techniques and treatment-related devices in RT.

Therefore, this study aimed to use the Survey of Medical Institutions Database to investigate the distribution of external RT facilities, the installation status of treatment-related devices and the implementation status of high-precision RT within Japan. The obtained data will be analyzed based on the hospital size and prefecture. In this analysis, the study also seeks to acquire specific and detailed information regarding the domestic development of RT by conducting trend investigations into the healthcare capabilities of treatment facilities.

## MATERIALS AND METHODS

### Study design and data sources

This retrospective study used data from the Survey of Medical Institutions and was approved by the Institutional Ethics Review Committee (2023-0-004). All studies were conducted by relevant guidelines and regulations.

The survey items included the number of facilities conducting external RT facilities, the number of facilities implementing high-precision RT (high-precision RT facilities), the number of external RT devices (RT devices) and the number of radiation treatment planning systems (TPS). The term ‘high-precision RT facilities’ refers to facilities that implement IMRT, stereotactic irradiation (STI) either or both. Additionally, the term ‘RT devices’ represents the total number of linear accelerators and gamma knives.

The results of the Survey of Medical Institutions are publicly available on the ministry’s website, and access to all data is free. The survey targets all hospitals with ≥20 beds, and the data are analyzed by categorizing them based on the number of hospital beds. Data from clinics with ≤19 beds are often undisclosed to prevent the identification of facilities, and because of their small overall percentage, they were excluded from the analysis. In the prefecture-wise analysis, an examination was conducted regarding the population and area densities. The population survey used the population estimation survey database from the Ministry of Internal Affairs and Communications Statistics Bureau [28], while the area survey employed the Geospatial Information Authority of Japan’s area database of the Ministry of Land, Infrastructure, Transport and Tourism [29].

### Detailed statistical analysis

Among the data obtained, a detailed analysis was added for the rate of high-precision RT implementation, the number of TPSs per RT device and the number of professionals involved in RT (certified radiation oncologists, radiological technologists, medical physicists, radiotherapy technologists and radiotherapy quality manager). The percentage of high-precision RT performed was calculated by dividing the number of high-precision RT facilities by the number of RT facilities, using data from the year 2020. The number of professionals involved in RT by prefecture was obtained from the respective certification organizations’ websites [5, 23, 30–32]. Specifically, we complied data on radiation oncologists (2022), radiological technologists (2020), medical physicists (2021), radiotherapy technologists (2023) and radiotherapy quality managers (2023). To investigate whether a statistical relationship exists between the percentage of high-precision RT performed and the number of TPSs per RT device or the number of professionals involved in RT, Spearman’s rank correlation coefficient was used. In the prefecture-wise analysis, Wilcoxon’s rank-sum test was conducted to perform statistical analysis on the changes between the initial and latest survey years. Then, the R software version 3.6.1 (The R Foundation for Statistical Computing, Vienna, Austria) was used for all statistical analyses. Statistical significance was set at *P* < 0.05.

## RESULTS

The Survey of Medical Institutions was conducted every 3 years from 1996 to 2020. Data for the number of RT facilities and devices were obtained from nine surveys conducted between 1996 and 2020. Data on the number of high-precision RT facilities and TPS were collected from five surveys conducted since 2008 until 2020. The data included a total of 6535 RT facilities, 7958 devices, 114 441 TPSs and 1429 high-precision RT facilities during the survey period.

### Transition of the number of facilities and devices

**Fig. 1:**
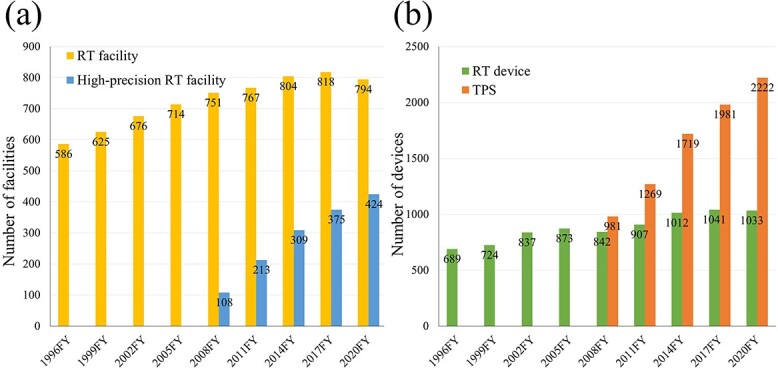
(**a**) Number of external RT facilities and high-precision RT facilities. (**b**) Number of external RT devices and TPSs.

Both the number of RT facilities and high-precision RT facilities showed an increasing trend from the initial survey year. Furthermore, the implementation rate of high-precision RT increased from 14% (108 facilities out of 751) in 2008 to 53% (424 facilities out of 794) in 2020 (Fig. 1a).

The number of RT devices and TPSs also exhibited an increasing trend, with the 2020 data showing 1033 devices and 2222 devices, respectively. Although the number of RT devices increased 1.5 times over 24 years (689 devices to 1033 devices), the number of TPSs increased more than doubled over 12 years (981 devices to 2222 devices) (Fig. 1b).

### Survey by the number of beds

**Fig. 2:**
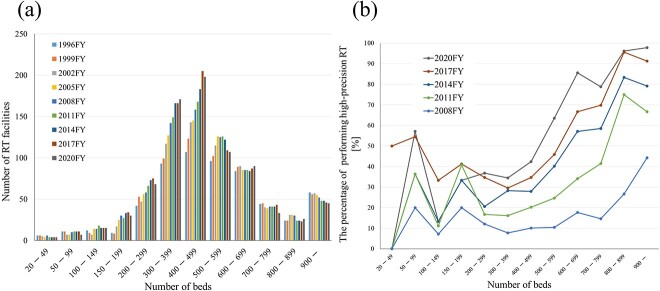
(**a**) Number of external RT facilities. (**b**) Percentage of performing high-precision RT by number of beds.

When compared with the initial survey year, facilities with 20–149 beds and 600–899 beds showed a relatively stable trend; however, those with ≥900 beds exhibited a declining trend. Intriguingly, a significant increase was observed in facilities with 150–599 beds (Fig. 2a); facilities with 300–399 beds increased 1.8-fold (from 93 to 171 facilities) and facilities with 400–499 beds increased 1.9-fold (from 107 to 198 facilities). The number of RT devices, TPSs and high-precision RT facilities showed a similar trend (Supplementary Figs 1a–c). Comparing data from all hospitals in 2020 revealed an RT implementation trend that increases as the number of beds increases (number of beds; 20–99, 100–199, 200–299, 300–399, 400–499, 500–599, 600–699, 700–799, 800–899, ≥900 and percentage of facilities performing RT; 0.4%, 1.6%, 6.6%, 25.3%, 53.7%, 66.5%, 81.1%, 78.6%, 92.9%, 86.5%, respectively; Supplementary Table 1).

In 2020, the percentage of facilities in Japan performing high-precision RT was 53.4% (Supplementary Table 1). The implementation rate of high-precision RT also increased in most hospital size categories, exceeding 95% in facilities with ≥800 beds (Fig. 2b). When limited to special functioning hospitals, 98.8% (85/86) of the total number of hospitals were performing RT, and 97.6% (83/85) of RT facilities were performing high-precision RT (Supplementary Table 1). However, in the 2020 data, only hospitals with >600 beds had a high-precision RT practice rate exceeding 70% (Fig. 2b). Facilities with 300–399 and 400–499 beds, which accounted for the largest number of RT facilities, accounted for 35% (59/171) and 42% (84/198) facilities both of which are below 50% (Supplementary Table 1).

**Fig. 3:**
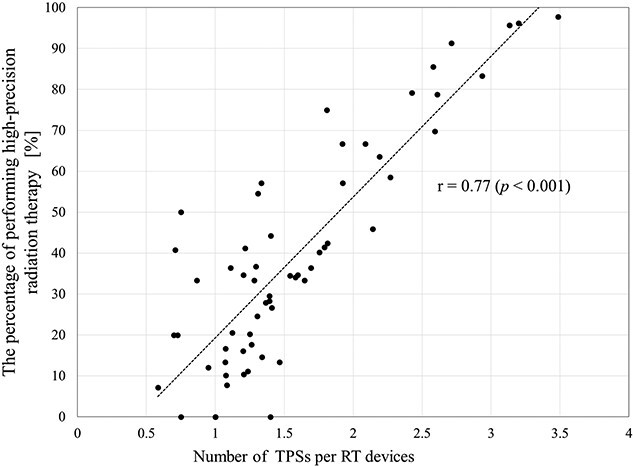
The relationship between the number of TPSs per RT devices and the percentage of performing high-precision radiotherapy.

Additionally, investigating the number of TPSs per RT device (Supplementary Fig. 2) revealed that facilities with 800 beds or more had three or more devices (2.4 times higher compared to 2008) and 600 beds or more had 2.5 or more devices. In contrast, facilities with 300–399 beds and 400–499 beds had 1.7 devices per RT device (1.6 times higher compared to 2008). A strong correlation (*r* = 0.77, *P* < 0.001) was observed between the number of TPSs per RT device and the percentage of high-precision RT performed (Fig. 3).

### Survey by prefecture

**Fig. 4:**
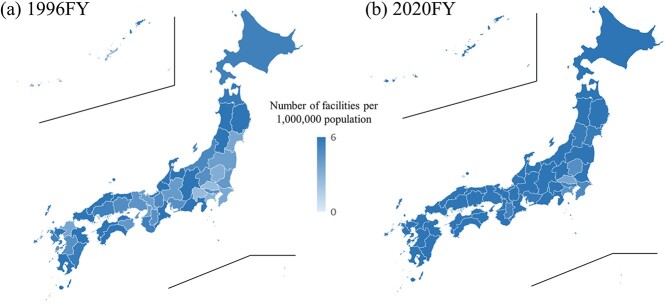
Number of RT facilities per 1 000 000 population by prefecture in (a) 1996FY and (b) 2020FY.

The number of RT facilities per 1 million population showed an increase in almost all prefectures, confirming a significant overall increase compared with the initial survey year (*P* < 0.001) (Fig. 4). The national average had increased from 4.7 in 1996 to 6.3 in 2020 (Supplementary Table 2). Furthermore, the ratio obtained by dividing the maximum (10.9, Tottori, 1996; 12.8, Tottori, 2020) by the minimum (2.5, Saitama, 1996; 3.5, Saitama, 2020) value of the number of RT facilities per 1 million population decreased from 4.4 (in 1996) to 3.7 (in 2020), indicating an improvement in regional disparities based on the population ratio (Supplementary Table 2).

**Fig. 5:**
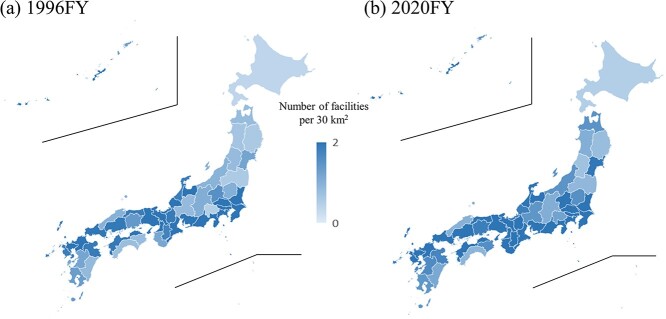
Number of RT facilities per 30 km^2^ by prefecture in (**a**) 1996FY and (**b**) 2020FY.

The number of treatment facilities per 30 km^2^ also significantly increased (*P* < 0.001) (Fig. 5). However, the ratio obtained by dividing the maximum (82.2, Tokyo, 1996; 95.1, Tokyo, 2020) by the minimum (0.9, Hokkaido, 1996; 1.3, Hokkaido, 2020) value (91.3 in 1996 vs 73.1 in 2020) was higher than the population ratio, indicating significant regional disparities in the density ratio (Supplementary Table 2).

**Fig. 6:**
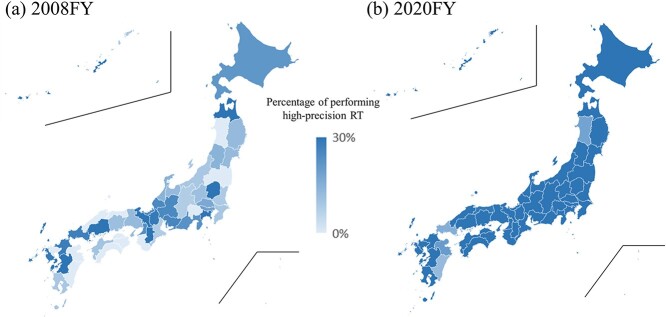
Percentage of performing high-precision RT by prefecture in (**a**) 2008FY and (**b**) 2020FY.

**Fig. 7:**
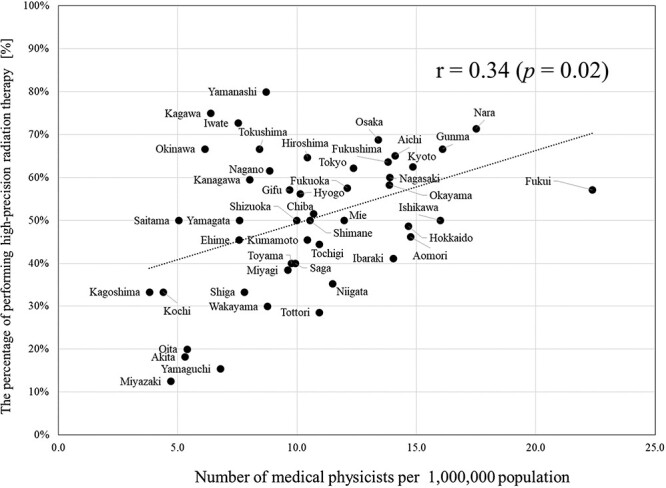
The relationship between the number of medical physicists per 1 000 000 population and the percentage of performing high-precision radiotherapy.

The high-precision RT implementation rate also showed an increasing trend (*P* < 0.001) (Fig. 6). In 2008, 12 prefectures (26%) were found to have a 0% implementation rate, but currently, all prefectures have implemented high-precision RT. Furthermore, the number of prefectures with a high-precision RT implementation rate of ≥50% increased from 1 prefecture (2%) in 2008 to 26 prefectures (55%) in 2020 (Supplementary Table 2). Regarding the correlation between the number of professionals involved in RT per 1 million population and the percentage of high-precision RT performed, a correlation was observed for medical physicists (r = 0.34, *P* = 0.02) (Fig. 7). However, no correlation was observed for radiation oncologists (r = 0.26, *P* = 0.08), radiological technologists (r = −0.16, *P* = 0.29), radiotherapy technologists (r = 0.05, *P* = 0.75) and radiotherapy quality manager (r = −0.03, *P* = 0.83) (Supplementary Fig. 3).

## DISCUSSION

### Transition of the number of facilities and devices

The number of RT facilities, high-precision RT facilities, RT devices and TPSs was confirmed to have an increasing trend. This can be attributed to the results of the government’s Basic Plan to Promote Cancer Control [33–35] and the proactive activities of related organizations [36]. In addition, the widespread adoption of high-precision RT is believed to be influenced by the expanded application of IMRT and STI in medical fees [37]. With regard to the increased number of facilities and devices, the results were similar to those of the JASTRO structural survey [5] and were corroborated by the results of the Survey of Medical Institutions, which had a response rate of almost 100%, and the consistency of the survey results was assured.

### Survey by the number of beds

Analysis of data from 1996 to 2020 by hospital size shows a broad division between facilities that have increased the implementation of RT and those that remained unchanged (or decreased slightly). The increase was particularly pronounced for facilities with 300–399 and 400–499 beds. Facilities with 300–499 beds had an RT implementation rate of 25.3–53.7% (Supplementary Table 1) compared to all hospitals. Since there was room for new RT to be initiated, further increases are expected in the future.

Regarding the implementation rate of high-precision RT, hospitals with ≥800 beds had a rate of ≥95%. These facilities include many university hospitals and cancer-specialized hospitals, and the high implementation rate is considered to be associated with the requirements for special functioning hospitals or designated cancer centers [38, 39]. Among hospital sizes where the implementation rate of high-precision RT is <70%, the largest number of hospitals belongs to the 400–499 beds category, followed by 300–399 beds. Future increases in the implementation rate of high-precision RT at facilities of these sizes will directly lead to a higher overall implementation rate of high-precision RT in Japan. For example, if the implementation rate of high-precision RT at hospitals with 300–499 beds reaches 70%, the overall percentage of performing high-precision RT in Japan will reach 68%, which closely aligns with the implementation rate overseas. These facilities include many that have recently started RT, and further increases are expected.

The number of TPSs per RT device strongly correlates with the high-precision RT implementation rate (r = 0.77). This confirms the current situation where many facilities have introduced multiple TPSs, considering the workload of high-precision RT and the extended time required during treatment planning [40].

### Survey by prefecture

The number of RT facilities per 1 million people was 6.3 on the national average in Japan. This surpasses the median (range) of high-income countries worldwide, which is 5.1 (0.4–11.6) [41] and is comparable to that of the USA (7.1) [42]. Moreover, globally, 57.4% of the total population is reported to live within approximately 30 km of an RT facility [43] (80% in the USA [42]), whereas in Japan, since the number of facilities per 30 km^2^ in 2020 was >1 in all prefectures, it can be considered as 100% coverage (Supplementary Table 2). Both population- and density-based ratios exceed the world average, indicating that Japan is in an environment where RT facilities are readily accessible. However, although more than 50% of patients in many advanced countries receive RT [44], this rate remains at 23.7% for the overall patient with cancer population in Japan [5]. Despite the population ratio disparity being 3.7 times in 2020 by prefecture, the density ratio disparity is substantial at 73.1 times. Additionally, the implementation rate of high-precision RT varies widely by prefecture, ranging from 18% to 80%. The short supply of radiation oncologists in Japan compared to other countries [45] is a contributing factor, along with the concentration of physicians in urban areas [46–48]. This underscores the need for future adjustments, such as increasing the number of radiation oncologists and sharing medical resources more effectively.

Furthermore, there was no correlation between the number of radiation oncologists per million population and the high-precision RT rate. Previous studies have reported correlations between the number of patients and the number of physicians as well as between the number of patients undergoing IMRT and the number of physicians [5, 49]. This difference could be explained by the fact that this study investigated the number of facilities rather than the number of patients. For example, facilities where multiple radiation oncologists perform IMRT and STI on multiple patients and facilities where a single neurosurgeon performs only STI are treated the same as a count ‘1’ in this study. In the Survey of Medical Institutions, high-precision RT techniques such as IMRT and STI, along with various devices like linear accelerators and gamma knives, were not distinguished. Despite differences in accreditation criteria based on the irradiation technique and equipment used, the fact that they were collectively treated together influenced the results. Conversely, the number of medical physicists per million people was found to correlate with the high-precision RT implementation rate. The workload for quality management to ensure patient safety and treatment quality has increased [50], heightening the significance of non-physician professionals. Among other professionals, a correlation was only observed for medical physicists. This could, in part, be because ‘engineer’ was explicitly defined as a ‘medical physicist, or radiotherapy quality managers etc.’ in the personnel requirements outlined in the appendix of medical fees, and many medical physicists are registered [51]. However, previous studies have reported a correlation between the number of non-medical physicist professionals and the number of high-precision RT performed [49], and further investigation, including staffing and educational environment [52], of radiotherapy departments may be necessary.

This study has some limitations: First, the Survey of Medical Institutions is based on data from October 1 every year, which includes variations such as hospital reorganizations and equipment updates. In addition, the prefecture-by-prefecture analysis does not consider the possibility that patients may have received the treatment in neighboring prefectures, which was not considered in this study. Second, complex factors, including facility requirements and medical fees for IMRT or STI, should be evaluated. In other words, the study results should be cautiously interpreted because the rate of high-precision RT implementation does not increase simply by increasing the number of TPSs per RT device or the number of medical physicists. Finally, as the data are from the Survey of Medical Institutions, detailed analysis is difficult for some questionnaire items. For example, IMRT or STI and linear accelerator or gamma knife cannot be separated. In addition, proton therapy facilities were not included in the analysis because they were excluded from the survey. It would be more appropriate to use the National Database of Health Insurance Claims and Specific Health Checkups Database (NDB) published by MHLW when investigating the number of cases by device [53, 54]. Detailed investigations may be challenging in large-scale surveys such as the Survey of Medical Institutions; therefore, research at the level of academic societies or research by the MHLW grants is expected [55].

In conclusion, using the Survey of Medical Institutions from the MHLW, we were able to elucidate trends in the number of external RT facilities and equipment in Japan. Detailed analysis by the number of beds and prefecture provided more specific information on the medical functions of RT facilities. The survey data are expected to contribute to the future development of radiotherapy, such as the equalization of treatment techniques and optimal allocation of medical resources.

## Supplementary Material

SupplementaryFigure1_rrae014

SupplementaryFigure2_rrae014

SupplementaryFigure3_rrae014

SupplementaryTable1_rrae014

SupplementaryTable2_rrae014
