# Supplementary material for: Trends and distribution of external radiation therapy facilities in Japan based on Survey of Medical Institutions from the Ministry of Health, Labour and Welfare
Source: J Radiat Res. 2024 Apr 11;65(3):328–36. doi: 10.1093/jrr/rrae014 (PMC11115472; doi:10.1093/jrr/rrae014)
Supplement: SupplementaryFigure1_rrae014 [file supplementaryfigure1_rrae014.docx]

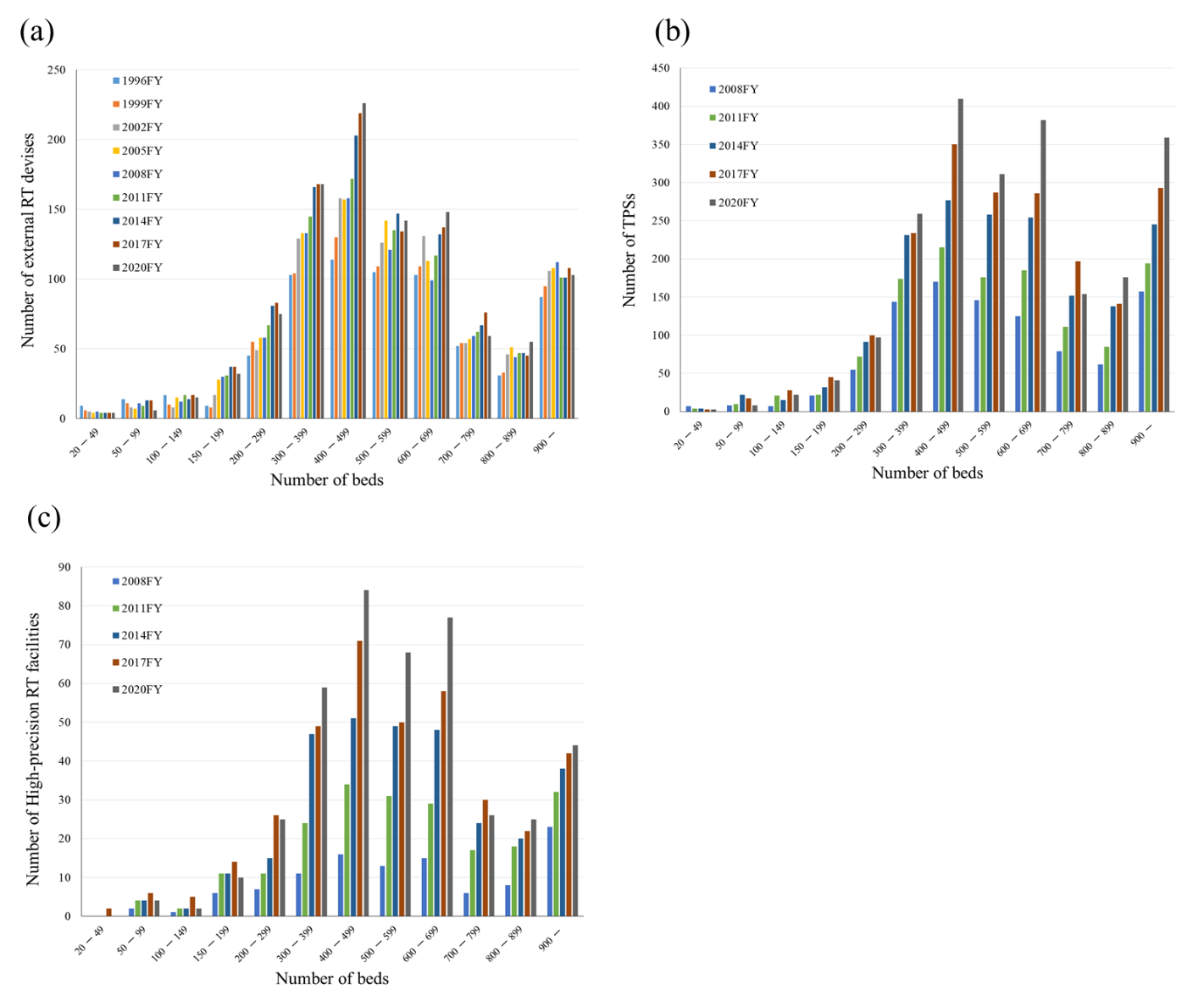


Supplementary Figure 1 (a) Number of external radiation therapy (RT) devices, (b) treatment planning systems, and (C) high-precision RT facilities by the number of beds.
