# Supplementary material for: Trends and distribution of external radiation therapy facilities in Japan based on Survey of Medical Institutions from the Ministry of Health, Labour and Welfare
Source: J Radiat Res. 2024 Apr 11;65(3):328–36. doi: 10.1093/jrr/rrae014 (PMC11115472; doi:10.1093/jrr/rrae014)
Supplement: SupplementaryFigure2_rrae014 [file supplementaryfigure2_rrae014.docx]

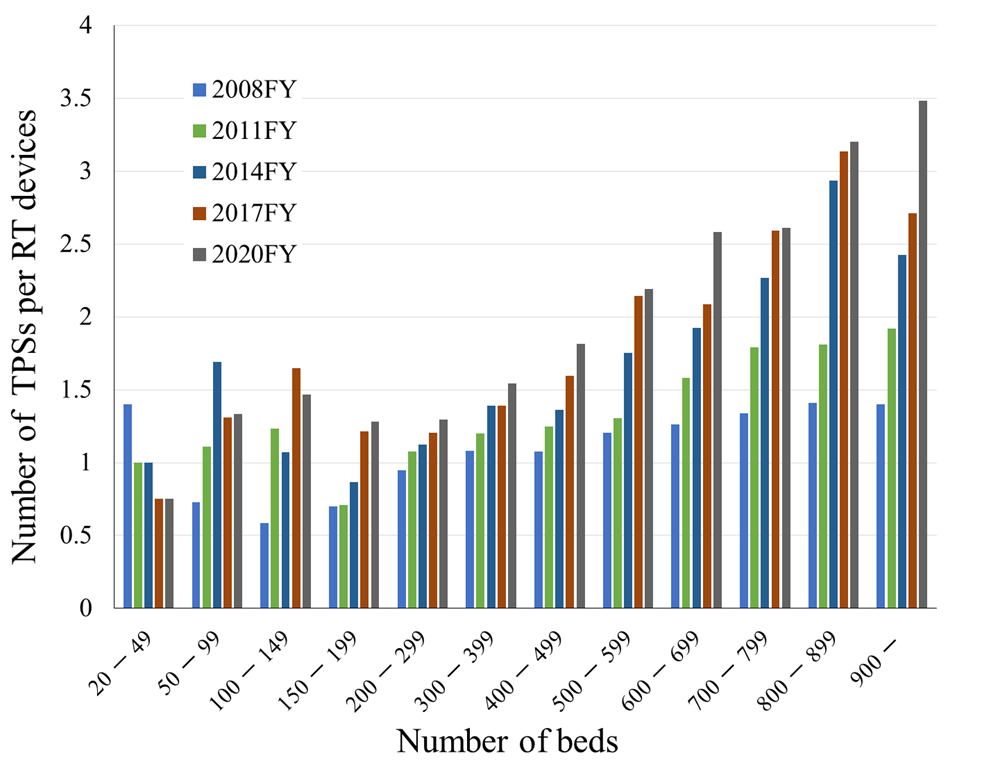


Supplementary Figure 2 Number of treatment planning systems (TPSs) per radiation therapy (RT) devices by the number of beds.
