# Supplementary material for: Trends and distribution of external radiation therapy facilities in Japan based on Survey of Medical Institutions from the Ministry of Health, Labour and Welfare
Source: J Radiat Res. 2024 Apr 11;65(3):328–36. doi: 10.1093/jrr/rrae014 (PMC11115472; doi:10.1093/jrr/rrae014)
Supplement: SupplementaryTable1_rrae014 [file supplementarytable1_rrae014.docx]

Supplementary Table 1 Number of hospitals and RT facilities by the number of beds or the type of hospital in 2020

| Number of beds or type of hospital | Total hospitals | RT facilities | High-precision RT facilities | Percentage of performing RT | Percentage of performing high-precision RT |
| --- | --- | --- | --- | --- | --- |
| 20－99 | 2970 | 11 | 4 | **0.4%** | 36.4% |
| 100－199 | 2792 | 45 | 12 | **1.6%** | 26.7% |
| 200－299 | 1036 | 68 | 25 | **6.6%** | 36.8% |
| 300－399 | 677 | 171 | 59 | **25.3%** | **34.5%** |
| 400－499 | 369 | 198 | 84 | **53.7%** | **42.4%** |
| 500－599 | 161 | 107 | 68 | **66.5%** | 63.6% |
| 600－699 | 111 | 90 | 77 | **81.1%** | 85.6% |
| 700－799 | 42 | 33 | 26 | **78.6%** | 78.8% |
| 800－899 | 28 | 26 | 25 | **92.9%** | 96.2% |
| 900－ | 52 | 45 | 44 | **86.5%** | 97.8% |
| Total | 8238 | 794 | 424 | 9.6% | **53.4%** |
| Special Functioning Hospitals | 86 | 85 | 83 | **98.8%** | **97.6%** |
| Non-Special Functioning Hospitals | 8152 | 709 | 341 | 8.7% | 48.1% |

Percentage of performing RT = RT facilities / Total hospitals

Percentage of performing high-precision RT = High-precision RT facilities / RT facilities
