# Supplementary material for: Trends and distribution of external radiation therapy facilities in Japan based on Survey of Medical Institutions from the Ministry of Health, Labour and Welfare
Source: J Radiat Res. 2024 Apr 11;65(3):328–36. doi: 10.1093/jrr/rrae014 (PMC11115472; doi:10.1093/jrr/rrae014)
Supplement: SupplementaryTable2_rrae014 [file supplementarytable2_rrae014.docx]

Supplementary Table 2 Number of facilities and the percentage of performing high-precision radiation therapy (RT) by prefecture

|  | RT facilities per 1,000,000 population | | Number of facilities per 30 km^2^ | | Percentage of performing high-precision RT | |
| --- | --- | --- | --- | --- | --- | --- |
| Survey year [FY] | 1996 | 2020 | 1996 | 2020 | 2008 | 2020 |
| Hokkaido | 5.4 | 7.1 | **0.9** | **1.3** | 21% | 49% |
| Aomori | 5.7 | 10.6 | 2.1 | 3.8 | 43% | 46% |
| Iwate | 6.7 | 9.2 | 1.5 | 2.0 | 13% | 73% |
| Miyagi | 3.5 | 5.7 | 3.1 | 5.0 | 13% | 38% |
| Akita | 7.4 | 11.6 | 1.7 | 2.7 | 0% | **18%** |
| Yamagata | 6.6 | 5.7 | 2.1 | 1.8 | 14% | 50% |
| Fukushima | 3.9 | 6.1 | 1.4 | 2.3 | 0% | 64% |
| Ibaraki | 4.2 | 6.0 | 5.6 | 7.9 | 8% | 41% |
| Tochigi | 3.1 | 4.7 | 2.6 | 4.0 | 33% | 44% |
| Gunma | 4.7 | 6.2 | 4.0 | 5.3 | 11% | 67% |
| Saitama | **2.5** | **3.5** | 13.4 | 19.3 | 17% | 50% |
| Chiba | 3.3 | 4.9 | 11.5 | 17.0 | 10% | 52% |
| Tokyo | 4.6 | 5.3 | **82.2** | **95.1** | 19% | 62% |
| Kanagawa | 2.9 | 4.5 | 31.6 | 49.1 | 30% | 60% |
| Niigata | 5.1 | 7.8 | 2.5 | 3.8 | 9% | 35% |
| Toyama | 7.8 | 9.8 | 5.3 | 6.7 | 13% | 40% |
| Ishikawa | 6.2 | 8.9 | 4.7 | 6.8 | 14% | 50% |
| Fukui | 10.5 | 9.2 | 5.4 | 4.7 | 25% | 57% |
| Yamanashi | 2.5 | 6.2 | 1.3 | 3.2 | 0% | **80%** |
| Nagano | 6.4 | 6.4 | 2.7 | 2.7 | 8% | 62% |
| Gifu | 4.1 | 7.1 | 2.1 | 3.7 | 25% | 57% |
| Shizuoka | 6.1 | 7.8 | 8.0 | 10.2 | 23% | 50% |
| Aichi | 4.7 | 5.7 | 19.1 | 23.5 | 26% | 65% |
| Mie | 7.4 | 6.8 | 6.4 | 5.9 | 8% | 50% |
| Shiga | 5.0 | 8.5 | 4.9 | 8.4 | 29% | 33% |
| Kyoto | 3.9 | 6.2 | 6.1 | 9.8 | 30% | 63% |
| Osaka | 3.9 | 6.9 | 50.4 | 90.5 | 24% | 69% |
| Hyogo | 4.4 | 5.9 | 8.1 | 10.8 | 13% | 56% |
| Nara | 4.6 | 5.3 | 4.6 | 5.4 | 33% | 71% |
| Wakayama | 5.5 | 10.9 | 3.0 | 6.0 | 0% | 30% |
| Tottori | **10.9** | **12.8** | 4.8 | 5.6 | 0% | 29% |
| Shimane | 9.0 | 9.0 | 2.5 | 2.5 | 0% | 50% |
| Okayama | 4.8 | 6.4 | 3.6 | 4.8 | 11% | 58% |
| Hiroshima | 5.4 | 6.1 | 5.0 | 5.7 | 47% | 65% |
| Yamaguchi | 8.3 | 9.8 | 5.1 | 6.0 | 27% | 15% |
| Tokushima | 4.2 | 8.4 | 2.0 | 4.1 | 0% | 67% |
| Kagawa | 9.6 | 8.5 | 13.6 | 12.0 | 0% | 75% |
| Ehime | 7.6 | 8.3 | 5.0 | 5.5 | 10% | 45% |
| Kochi | 7.3 | 8.8 | 2.0 | 2.4 | 0% | 33% |
| Fukuoka | 3.5 | 6.4 | 10.2 | 18.7 | 28% | 58% |
| Saga | 6.2 | 6.2 | 5.8 | 5.8 | 0% | 40% |
| Nagasaki | 5.4 | 7.7 | 4.8 | 6.8 | 29% | 60% |
| Kumamoto | 4.6 | 6.4 | 3.1 | 4.2 | 38% | 45% |
| Oita | 9.9 | 9.0 | 4.9 | 4.5 | 0% | 20% |
| Miyazaki | 5.7 | 7.5 | 2.2 | 2.9 | 0% | 13% |
| Kagoshima | 7.0 | 7.6 | 3.4 | 3.7 | 9% | 33% |
| Okinawa | 2.7 | 6.1 | 5.0 | 11.1 | 75% | 67% |
| Total | 4.7 | **6.3** | 4.4 | 5.9 | 18% | 53% |
